# Pharmacological profiles of opioid ligands at Kappa opioid receptors

**DOI:** 10.1186/1471-2210-6-3

**Published:** 2006-01-25

**Authors:** Parham Gharagozlou, Ezzat Hashemi, Timothy M DeLorey, J David Clark, Jelveh Lameh

**Affiliations:** 1 Department of Pharmacology, Molecular Research Institute, Mountain View, CA 94043, USA; 2 Department of Anaesthesiology, VA Palo Alto Health Care System, Palo Alto, CA, 94034, USA; 3 ACADIA Pharmaceuticals Inc., San Diego, CA 92121, USA

## Abstract

**Background:**

The aim of the present study was to describe the activity of a set of opioid drugs, including partial agonists, in a human embryonic kidney cell system stably expressing only the mouse κ-opioid receptors. Receptor activation was assessed by measuring the inhibition of cyclic adenosine mono phosphate (cAMP) production stimulated by 5 μM forskolin. Intrinsic activities and potencies of these ligands were determined relative to the endogenous ligand dynorphin and the κ agonist with the highest intrinsic activity that was identified in this study, fentanyl.

**Results:**

Among the ligands studied naltrexone, WIN 44,441 and dezocine, were classified as antagonists, while the remaining ligands were agonists. Intrinsic activity of agonists was assessed by determining the extent of inhibition of forskolin-stimulated cAMP production. The absolute levels of inhibition of cAMP production by each ligand was used to describe the rank order of intrinsic activity of the agonists; fentanyl = lofentanil ≥ hydromorphone = morphine = nalorphine ≥ etorphine ≥ xorphanol ≥ metazocine ≥ SKF 10047 = cyclazocine ≥ butorphanol > nalbuphine. The rank order of affinity of these ligands was; cyclazocine > naltrexone ≥ SKF 10047 ≥ xorphanol ≥ WIN 44,441 > nalorphine > butorphanol > nalbuphine ≥ lofentanil > dezocine ≥ metazocine ≥ morphine > hydromorphone > fentanyl.

**Conclusion:**

These results elucidate the relative activities of a set of opioid ligands at κ-opioid receptor and can serve as the initial step in a systematic study leading to understanding of the mode of action of these opioid ligands at this receptor.

## Background

Opioid ligands possess a variety of physiological activities and medical uses, with the most prominent being in the treatment of pain. Pharmacological studies indicate that selective μ-opioid agonists are effective antinociceptive agents in virtually every test of analgesia [1,2]. However, at their analgesic doses, μ-opioid receptor agonists can induce ventilatory depression [3] and the development of physical dependence [4]. Delta opioid receptors have been reported to modulate analgesia, autonomic nervous system function, neuroendocrine system function, and mood driven behaviors [5]. Activity of κ-opioid receptors modulate spinal antinociception [6]. Mu and κ – but not δ-opioid receptors modulate ventilatory depression [7]. Thus each class of opioid receptors represents an important drug target to investigate.

A major medical application of opioid ligands has been as potent analgesics. However, untoward effects associated with opioids limit their wider use for analgesia. Numerous opioid ligands have been synthesized with the promise of effective analgesia and minimal side-effects; however this goal has yet to be realized. The studies leading to the synthesis of novel opioid ligands have relied on research in animals or tissues expressing multiple opioid receptors. Thus, characterization of the activity profiles for these opioid ligands at individual opioid receptors has only been possible after the cloning of opioid receptors. Such information is essential to the design of a new generation of opioid analgesics that would exhibit diminished side-effects.

We have previously characterized fifteen opioid ligands in cells expressing only δ-opioid receptor [8] or μ-opioid receptor [9]. The present study was devised to characterize the activity of these same opioid ligands in a cell line expressing only κ-opioid receptors. The ligands were chosen based on our previous model tissue data suggesting that they bind to all three opioid receptor types [10] and some display differential activation profiles *in vivo *at each of the opioid receptor types [11]. Thus, the present study was designed to achieve the following goals; (1) to describe the activation profiles of a set of opioid ligands not previously defined in an isolated cell system expressing only κ-opioid receptor using inhibition of forskolin-stimulated adenylyl cyclase activity in intact cells, and (2) to compare the intrinsic activities of these drugs to the known κ-opioid receptor agonist with very high intrinsic activity, fentanyl, and the endogenous κ-opioid ligand, dynorphin. The results obtained from this study can serve to clarify the categorization of each of the ligands studied as an agonist, weak/partial agonist or antagonist at κ-opioid receptors. Moreover, these results demonstrate the interaction of each drug with a single receptor type at the molecular level. Finally, these results, together with our previously published data on these ligands at μ and δ opioid receptors, help define the activity of these ligands at all three opioid receptor types.

## Results

The binding affinity and activation potency of fifteen opioid ligands were assessed in HEK cells stably expressing κ-opioid receptors.

### Binding assays

To further investigate the activity or affinity of selected ligands at κ-opioid receptors, competition binding assays against a radiolabeled ligand were performed using cell homogenates from transfected cells. Human embryonic kidney (HEK) cells were transfected with mouse cDNA for κ-opioid receptor. These cells do not normally express endogenous κ-opioid receptors, as demonstrated by a lack of binding to the radioactive [^3^H]-U69,593 (data not shown). Individual HEK clones expressing single opioid receptor types were propagated and used for the experiments presented here. Expression level of the selected clone was 492 ± 39 fmole/mg protein. Similar levels of expression have been reported for these receptors in neurons [12] and for other transfected cells expressing this receptor [13]. Competition binding studies were carried out for each ligand in the presence of [^3^H]-U69,593. All ligands studied exhibited K_i _values in the nanomolar range. The rank order of affinity of these ligands was; cyclazocine > naltrexone ≥ SKF 10047 ≥ xorphanol ≥ WIN 44,441 > nalorphine > butorphanol > nalbuphine ≥ lofentanil > dezocine ≥ metazocine ≥ morphine > hydromorphone > fentanyl.

### Activation assays

**Figure 1:**
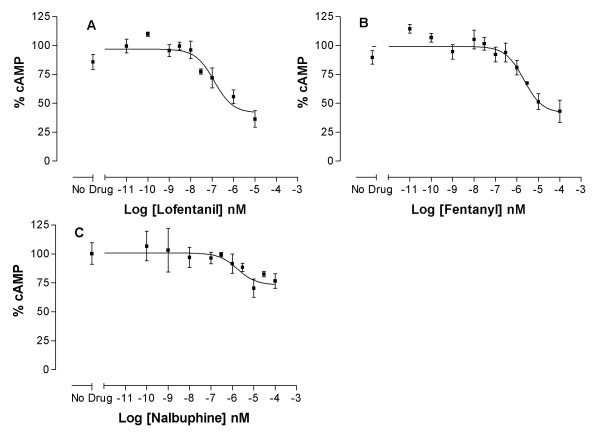
**Dose response curves of inhibition of adenylyl cyclase activity by representative ligands in HEK-κ cells**. Varying concentration of 2 full agonists (lofentanil and fentanyl) and one partial agonist (nalbuphine) were used to determine the potency and intrinsic activity of each ligand in inhibiting the effect of 5 μM forskolin in producing cAMP, as described under methods. The 100% on the y-axis corresponds to the cAMP levels in the absence of any drug, i.e.: forskolin alone, for all figures. Data presented are the average from 2 or more experiments carried out in duplicate. Error bars represent standard error of the mean (SEM) of the normalized data. Data have been normalized and SEM calculated as described under methods. (A) Full agonist lofentanil, (B) Full agonist, fentanyl, (C) Partial agonist, nalbuphine.

**Figure 2:**
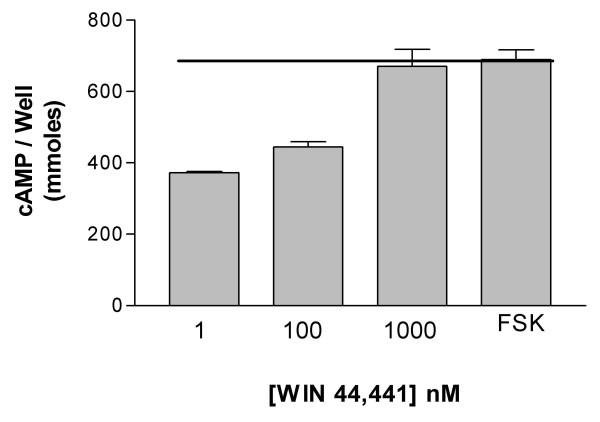
**Antagonism of inhibition of adenylyl cyclase activity by a representative ligand in HEK-κ cells**. Reversal of the inhibitory effect of etorphine by the antagonist Win 44,441 is shown. Maximal cAMP levels were in the range of 400–1000 pmole/well. Data presented are the average from 2 or more experiments carried out in duplicate. Error bars represent standard error of the mean.

Overall, a range of intrinsic activities and potencies were observed for the different ligands at κ-opioid receptor (Table 1). Butorphanol, cyclazocine, etorphine, fentanyl, hydromorphone, lofentanil, metazocine, morphine, nalorphine, SKF 10047 and xorphanol were full agonists. These ligands inhibited forskolin-stimulated cAMP production to the levels that were not significantly different from the inhibitory levels of the endogenous ligand dynorphin. Naltrexone, dezocine and WIN 44,441 behaved as an antagonist (Fig. 1). These ligands exhibited little or no measurable inhibitory effect on forskolin-stimulated cAMP production when used alone and were able to block the inhibitory effect of 1 nM etorphine (IC_50 _= 2 nM, 8.5 μM and 300 nM respectively). Finally, nalbuphine exhibited partial agonist activity at these receptors. Statistical analysis of the differences between the intrinsic activities of different ligands was carried out to distinguish the full agonists from the partial agonists. Based on these analyses, there was no difference in the intrinsic activity of any of the agonists compared to both dynorphin and fentanyl (the ligand with the highest intrinsic activity, Fig. 1A), except for nalbuphine, which had significantly lower intrinsic activity (p < 0.05). Thus, nalbuphine is a true partial agonist at κ-opioid receptors (Fig. 1C). Nevertheless, if these ligands were to be ranked based on their absolute level of activity, irrespective of statistical significance, their rank order of intrinsic activity would be fentanyl = lofentanil ≥ hydromorphone = morphine = nalorphine ≥ etorphine ≥ xorphanol ≥ metazocine ≥ SKF 10047 = cyclazocine ≥ butorphanol > nalbuphine. Finally, as seen in Figure 2, 100 nM Win 44,441 could antagonize the effect of etorphine, and 1 μM of WIN 44, 441 completely antagonized the effect of 1 nM etorphine.

**Table 1:** Binding affinity, potency, and intrinsic activity of opioid ligands in inhibiting adenylyl cyclase activity.

**Ligands**	**EC_50 _± SEM (nM)**	**% Max Inhibition (Mean +/- SEM)**	**Relative Intrinsic activity**	**HEK-κ Ki (nM)**
**Butorphanol**	57 ± 47	33 ± 7	0.57	2.5 ± 0.8
**Cyclazocine**	2 ± 2	39 ± 5	0.67	0.1 ± 0.0
**Dezocine**	Antagonist	---------	Antagonist	24.5 ± 1.5
**Etorphine**	0.4 ± 0.3	52 ± 4	0.90	ND
**Fentanyl**	1677 ± 917	58 ± 9	1.00	233 ± 33
**Hydromorphone**	279 ± 135	55 ± 6	0.95	55 ± 17
**Lofentanil**	153 ± 76	58 ± 6	1.00	8.2 ± 1.9
**Metazocine**	56 ± 13	47 ± 5	0.81	24 ± 7.5
**Morphine**	213 ± 137	55 ± 5	0.95	26 ± 3
**Nalbuphine**	2550 ± 1759	27 ± 7	0.47 *	6 ± 1
**Nalorphine**	483 ± 245	55 ± 7	0.95	1.6 ± 0.1
**Naltrexone**	Antagonist	--------	Antagonist	0.3 ± 0.1
**SKF 10047**	24 ± 6	38 ± 4	0.66	0.4 ± 0.2
**Win 44441**	Antagonist	--------	Antagonist	0.5 ± 0.2
**Xorphanol**	3.3 ± 2	49 ± 4	0.84	0.4 ± 0.2

Effective concentrations of opioid ligands in inhibiting forskolin-stimulated adenylyl cyclase activity were measured as described under "Materials and Methods". Data for EC_50_'s represent the mean ± SEM obtained from two or more experiments carried out in duplicate. Maximum inhibition data represent the mean ± SEM obtained from the best fit curve for data from two or more experiments carried out in duplicate. All compounds denoted as antagonists completely reversed the effect of 1 nM etorphine. The effect of 1 nM etorphine in inhibiting cAMP production was 50–60% of maximum etorphine effect. Lofentanil and fentanyl had the highest intrinsic activity among the ligands tested. Relative intrinsic activity of the ligands were also compared to that of the putative endogenous ligand for κ-opioid receptor, dynorphin (EC_50 _= 8 ± 2 nM, intrinsic activity = 52 ± 3%). All three ligands, lofentanil, fentanyl and dynorphin had similar intrinsic activity. The intrinsic activity of each ligand relative to fentanyl (designated as 1) and the endogenous ligand dynorphin was determined. Statistical significance was calculated for the inhibitory effect of each ligand in comparison to fentanyl and dynorphin. (*). Statistically significant difference (*p *< 0.05) relative to fentanyl or dynorphin suggested that the test ligand was a partial agonist compared to both reference ligands. The binding affinity of each ligand was measured by competition assays. The Ki for each ligand in denoted in the table.

ND = Not determined

* = p < 0.05

## Discussion

A complex interaction between various opioid receptors exists as they modulate various physiological functions. Understanding these complex interactions requires a thorough understanding of the activation pattern of individual opioid receptors. Available data on the activation profiles of the test ligands relative to each specific opioid receptor were found to be either sparse or involved various systems and cell lines, thus making meaningful comparisons between affinities, intrinsic activities and potencies across various studies unreliable. The present study utilized a cell system that allowed for the expression of κ-opioid receptor subtype. Previously, these ligands were characterized in the same expression system expressing μ or δ-opioid receptors [8,9]. Thus, this data combined with the data reported in the last two reports can be used to compare the relative selectivity of these same ligands at the three different opioid receptors expressed in the same cell system.

A well-defined receptor expression system was used to characterize the activity of a set of opioid ligands and to compare the intrinsic activities and potencies of these ligands at κ-opioid receptors. The main advantage of using transfected HEK cells for this study is that these cells do not contain endogenous κ-opioid receptors, but express the G proteins necessary for the proper coupling of the transfected cDNAs to the respective second messenger systems. A well established method for assessing G-protein activation by opioid receptors and characterizing activity of opioid ligands is measuring the extent of inhibition of forskolin-stimulated adenylyl cyclase activity [14-19]. Adenylyl cyclase has been implicated in playing a role in mediating the analgesic effect of opioid ligands through μ-opioid receptors [20-22]. Thus, characterizing the ability of opioid ligands to inhibit cAMP production, such as described in this report, could be used as an index for assessing their activity as a correlate of their analgesic effects. Moreover, a simple well-defined system, such as the one used in this study, can be very beneficial in describing the mode of action of each ligand at a specific receptor. Although HEK cells are not neuronal in nature, and might be devoid of some of the intracellular machinery present in the body, nevertheless they serve as a useful tool for such studies on a single receptor. Furthermore, it is understood that once applied to the whole animal *in vivo*, the overall effect of the drug will be a composite of the effects of the drug on all receptor types interacting with it. In addition, the pharmacokinetic parameters, such as metabolism, tissue absorption and distribution of the drug will play a major role in the overall drug effect *in vivo*. The present set of ligands exhibited a range of intrinsic activities and potencies. The drug with the highest intrinsic activity was fentanyl, supporting previous *in vivo *studies describing fentanyl as a highly potent and efficacious analgesic drug [11]. In the present study, the intrinsic activity of lofentanil was equal to that of fentanyl. Nalbuphine was the only ligand that showed significantly lower intrinsic activity compared to fentanyl and dynorphin. The low intrinsic activity of this ligand combined with its high binding affinity to κ-opioid receptors (6 nM) suggest that nalbuphine can potentially act as competitive antagonists at the κ-opioid receptors *in vivo*. While the EC_50 _of this compound in inhibiting cAMP production is in micromolar range, based on the nanomolar binding affinity, the concentration of this compound required to block the activity of agonists (IC_50_) *in vivo *could be much lower.

## Conclusion

In summary, this report describes a detailed comparative study of the binding affinities and inhibitory effects of a set of opioid ligands on the accumulation of cAMP in intact cells expressing κ-opioid receptors. The intrinsic activities of these ligands have been compared to that of the endogenous opioid ligand, dynorphin, and the ligand identified in this study as having the highest intrinsic activity, fentanyl. Moreover, this report serves to clarify the activity of many previously un-characterized ligands in cells expressing only κ-opioid receptors, thus leading to a better understanding of the mechanism of action of these drugs.

## Methods

### Cell culture

Human Embryonic Kidney (HEK) 293 cells were maintained in D-MEM/F-12 (Dulbecco's Modified Eagle's Medium: Nutrient Mixture F-12 1:1 mixture), supplemented with 10% (v/v) fetal calf serum (FCS), 200 μg/ml G-418 (Geneticin^®^) in a humidified incubator with 5% CO_2 _and 95% air, at 37°C. The incubation medium was changed every 3–4 days. Once a week, cells were re-plated at 20% density into 75 cm^2 ^tissue culture flasks.

### Establishing stable cells expressing κ-opioid receptors

HEK 293 cells were transfected with the cDNA for mouse κ-opioid receptor using the lipofectin^® ^reagent (Life Technologies, Rockville, MD). Mouse κ-opioid receptor cDNA was in the vector pCMV (a generous gift from Dr. G. Bell) and was co-transfected with the vector pRSVneo in order to establish a stable clone. Stable clones were selected using 400 μg/ml Geneticin^®^. A single clone expressing 492 ± 39 fmole/mg protein for κ-opioid receptors as assessed by [^3^H]-U69-593 binding was selected for these studies.

### Binding assays

Saturation and competition binding assays were carried out in HEK-κ cells using [^3^H]-U69-593. Experiments were carried out as described previously [9,23]. Each assay was carried out in triplicates in a 250 μl total reaction volume containing 20–25 μg of crude cell homogenate per assay tube. Incubation was in 50 mM Tris HCl buffer, pH 7.4 at room temperature for 2 hours. The assay was terminated by rapid filtration through Whatman GF/B filters followed by three washes, with ice-cold buffer. Radioactivity retained on the filters was measured using liquid scintillation counting.

Competition binding assays were carried out in crude homogenate of HEK-κ cells. Binding was carried out in 250 μl volume of 50 mM Tris HCl buffer, pH 7.4 in the presence of about 0.5–1 nM [^3^H]-U69-593 and increasing concentrations (24–32) of unlabeled ligand. Incubation and washing were as described above. Binding data were analyzed using PRISM™ software. The values determined using these two analysis methods were in agreement. For preparation of crude cell homogenate, confluent cultures of HEK-κ cells were harvested using phosphate buffered saline. Following centrifugation, the cell pellet was re-suspended in ice-cold 50 mM Tris HCl buffer pH 7.4 at about 10^7^cell/ml, and homogenized using a polytron at setting 6 for 10 seconds. The cell homogenate was stored in aliquots at -86°C until use. Protein content of the cell homogenate was determined using Bio-Rad protein assay reagent (Bio-Rad, Hercules, CA).

### Whole cell adenylyl cyclase assays

Inhibition of forskolin-stimulated cyclic AMP (cAMP) production after exposure to each ligand was evaluated in intact transfected HEK cells as reported previously [9]. Exponentially growing transfected HEK cells were harvested and re-suspended in serum free DMEM/F12 medium. Cells were plated in 96 well microtiter plates at 5 × 10^4 ^cells/well in 100 μl volume. To each well, phosphodiesterase inhibitor 3-isobutyl-1-methylxanthine (IBMX) was added to a final concentration of 100 μM, followed by addition of agonists at different concentrations and incubation at 37°C. Following incubation for 15 minutes, forskolin was added to each well to a final concentration of 5 μM followed by another incubation for 15 minutes at 37°C. The EC_50 _of forskolin in these cells was 10 μM [24]. The reaction was terminated by aspiration of the medium and addition of lysis buffer from the Biotrak™ cAMP Enzyme Immunoassay kit from Amersham Pharmacia Biotech (Buckinghamshire, England). The rest of the assay followed the protocol provided with the kit. Actual amount of cAMP was determined for each sample in comparison to a standard curve of known amounts of cAMP provided in the cAMP kit, as described in the kit protocol.

### Agonism

Agonistic activity of opioid ligands was assessed by measuring the inhibitory effect of the drugs on forskolin-stimulated cAMP accumulation. Data were normalized to the top of the curve (no drug, 100%), expressed as percent inhibition of forskolin-stimulated cAMP accumulation and were fitted to a sigmoidal function by using one site competition function as described below. The intrinsic activity of each ligand was defined as percent inhibition of forskolin-stimulated cAMP production compared to no drug levels (0% inhibition, 100% cAMP production).

### Antagonism

Compounds with no or very small *in vitro *agonistic activity (<20% inhibition of cAMP production) for which the dose response curves could not be fitted due to the small effect, were tested for antagonism. Antagonists were defined as ligands that were able to block the inhibitory effect of 1 nM etorphine on forskolin-stimulated cAMP production. In these assays, antagonists were added to the cells along with IBMX. After 15 minutes of incubation, the agonist was added and the cells were incubated with both drugs for an additional 10 minutes. The rest of the assay was as described above.

### Curve fitting

The analysis of drug activity was performed using PRISM™ software (GraphPad Software, Inc. San Diego, CA). A computer-generated "best fit" of non-linear regression data was used to provide an estimate of the effective concentration at 50% (EC_50_). Dose response data generated by cAMP enzyme immunoassay (EIA) system were fitted to the one site competition function.

### Data processing

Data from each dose response curve were normalized to the top of the respective curve. The normalized data from multiple independent dose response curves were combined and a new dose response curve was fitted to the combined data and the EC_50 _and maximal inhibition were determined for the combined data. In all cases standard error of the mean (SEM) of multiple measurements was calculated using the formula; SEM = sd/√n, where sd = standard deviation and n = number of observations.

### Drugs

Forskolin, fentanyl, IBMX, hydromorphone, and naltrexone hydrochloride were obtained from Sigma-Aldrich (St. Louis, MO), nalbuphine was obtained from RBI (Natik, MA), cyclazocine, etorphine, metazocine, morphine sulfate, nalorphine, and SKF 10047 were obtained from National Institute of Drug Abuse (Bethesda, MD), lofentanil was from Janssen Pharmaceutical Inc. (Titusville, NJ), dezocine was from Wyeth Laboratories (Philadelphia, PA), Win 44,441 was from Sterling Winthrop Pharmaceutical and xorphanol was from Miles Inc. Pharmaceutical Division (West Haven, CT). All tissue culture reagents were purchased from Life Technologies (Rockville, MD). [^3^H]-U69, 593 was from Multiple Peptide Systems (San Diego, CA). All other reagents were of analytical grade from standard commercial sources.

All ligands used were prepared as 10 mM stock solutions in water except WIN 44,441, which was 5 mM. All ligands were dissolved in distilled water except cyclazocine, dezocine, and etorphine, which were dissolved in 100% ethanol. For the drugs dissolved in ethanol, the final concentration of ethanol in the reaction was <0.01% which had no affect on the assays performed.

### Statistical analysis

Maximal inhibitory effect of each ligand was compared to the levels of maximal inhibition by dynorphin and fentanyl using ANOVA analysis with Dunnett's multiple comparison as post-test using PRISM™ software (GraphPad Software, Inc. San Diego, CA). Significant difference between the inhibitory effects of two ligands was determined whenever p < 0.05.

## Authors' contributions

Author 1 (P.G.) carried out the cAMP assays, performed data and statistical analysis and drafted the manuscript. Author 2 (N.H.) performed some of the receptor binding assays. Authors 3 and 4 (T.M.D & J.D.C.) provided intellectual input and critical interpretation of the data. Author 4 (J.L.) conceived of the study, participated in its design and coordination, carried out most of the binding assays and finalized the manuscript for publication.
